# Intramuscular Adipose Tissue, Sarcopenia, and Mobility Function in Older Individuals

**DOI:** 10.1155/2012/629637

**Published:** 2012-02-06

**Authors:** Robin L. Marcus, Odessa Addison, Leland E. Dibble, K. Bo Foreman, Glen Morrell, Paul LaStayo

**Affiliations:** ^1^Department of Physical Therapy, University of Utah, 520 Wakara Way, Salt Lake, UT 84108, USA; ^2^Department of Radiology, University of Utah, 30 N 1900 E, Salt Lake, UT 84132, USA

## Abstract

*Objective*. Intramuscular adipose tissue (IMAT) and sarcopenia may adversely impact mobility function and physical activity. This study determined the association of locomotor muscle structure and function with mobility function in older adults. *Method*. 109 older adults with a variety of comorbid disease conditions were examined for thigh muscle composition via MRI, knee extensor strength via isometric dynamometry, and mobility function. The contribution of strength, quadriceps lean tissue, and IMAT to explaining the variability in mobility function was examined using multivariate linear regression models. *Results*. The predictors as a group contributed 27–45% of the variance in all outcome measures; however, IMAT contributed between 8–15% of the variance in all four mobility variables, while lean explained only 5% variance in only one mobility measure. *Conclusions*. Thigh IMAT, a newly identified muscle impairment appears to be a potent muscle variable related to the ability of older adults to move about in their community.

## 1. Introduction

The European Working Group on Sarcopenia in Older People [1] has recently suggested that the definition of sarcopenia should include not only low muscle mass but also low muscle function (strength or performance) because sarcopenia is a syndrome that is characterized by both. The loss of muscle mass and strength are problems that plague many older adults, though the causal and correlative link between diminishing lean mass and strength is not well supported in aging humans [2]. Aging is also characterized by increasing depots of fat both between and within skeletal muscles [3]. This increase in intramuscular adipose tissue (IMAT), and its lipotoxic effect, has been identified as a potential contributor to declining strength and muscle quality; with some also associating it with mobility limitations in older adults [4–8].

 The recent structural focus on IMAT content rather than lean muscle mass is important as it could provide a plausible explanation for age-associated strength and mobility deficits. Fatty infiltration into skeletal muscle may alter muscle fiber orientation and hence the force producing capabilities of the whole muscle. IMAT may also be a metabolically active component of muscle that secretes inflammatory cytokines leading to systemic inflammation, much like visceral adipose tissue, that can inhibit muscle force production even in the absence of muscle atrophy [9]. Further, IMAT may be an appropriate therapeutic rehabilitation target as some suggest its presence can blunt the strength response to resistance training [10].

 Currently, it is accepted that muscle weakness, loss of lean tissue mass, and IMAT contribute to functional decline. In order to gain insight and parcel out the contributions of these muscle variables to mobility, this study examined the association of IMAT of the thigh, along with muscle lean tissue and muscle strength, with mobility measures in older adults.

## 2. Methods

### 2.1. Subjects

One hundred and nine older (mean = 74.1 years ± 6.8) adults participated in this study. All were participants in exercise intervention studies at the University of Utah. The subjects' preexercise status was used in this paper. Recruitment occurred over a 5-year period from 2006–2011 and consisted of receiving names and identifying information from clinical databases at the University of Utah. Each of the subjects received a personal letter providing information about the study, and they were then contacted directly via phone or in-person to assess their interest and to screen for eligibility following receipt of a signed informed consent document. The 109 subjects were community ambulating males (*n* = 32) and females (*n* = 77) 60 years of age or older with 2 or more comorbid disease conditions and were at risk for falling due to fatigue, muscle weakness and/or had experienced a fall in the previous 12 months. All scored >23 on a Folstein minimental examination or passed the mini-Cog instrument for dementia. Individuals were excluded if they had any of the absolute contraindications for MRI, progressive diagnosed neurologic disease (e.g., Parkinson's, multiple sclerosis, Guillain-Barre, and Alzheimer's), any dystrophies, or rheumatologic conditions that primarily affect muscle (muscular dystrophy, PMR) or had been participating in regular (3x/week) aerobic or resistance exercise over the past 12 months (Table 1). 

### 2.2. Procedures

#### 2.2.1. Muscle Strength

Knee extension strength was determined via a maximum voluntary isometric contraction (MVIC) on a KinCom dynamometer (Chattanooga Inc., Hixon, TN) as follows: participants were stabilized by chest and thigh straps and seated with their knees fixed at 60 degrees of flexion with their arms folded across their chest. Prior to testing, participants practiced submaximal contractions at 50 and 75% of their perceived maximal effort prior to one practice maximal contraction trial. After a 2-minute rest period, three separate maximal contractions were performed. Each maximal contraction was held for 5 seconds with a 3-minute rest between trials. The outcome variable muscle force was calculated as the average peak force of three trials. The order of testing (right versus left) was randomized among subjects. Muscle strength was normalized by dividing peak muscle force (N) by BMI.

#### 2.2.2. Muscle Size and Composition

Bilateral magnetic resonance imaging (MRI) scans of the thighs were obtained. The respective IMAT and lean tissue cross-sectional areas were calculated from the MRI scans. Subjects were placed supine in a 3.0 Tesla whole body MR imager (Siemens Trio, Siemens Medical, Erlangen, Germany). The legs were scanned in a coronal plane with a turbo spin echo (TSE) T_1_-weighted sequence to depict the femoral heads and the femoral condyles. The midpoint of the thigh was determined and defined as half way between the superior margin of the femoral head and the inferior margin of the femoral condyles. Axial imaging (5 mm thick slices at 1 cm intervals) of the legs was then performed over 1/2 the length of the femur, centered at the midpoint of the thigh. This was performed with a three-point Dixon multislice 2D gradient recalled echo (GRE) sequence (TR = 300 ms, TE = 5.15/6.4/7.65 ms, and matrix size = 512 × 288). The three echo time acquisitions were acquired in separate sequence repetitions. Field of view was adjusted to the individual subject anatomy to obtain optimal in-plane spatial resolution, typically 1 × 1 mm or better. Separate fat and water images were created with custom software using the three-point Dixon method. A tissue model was then used to calculate estimates of total fat and nonfat volume fractions on a per-pixel basis, which were displayed in image form. All sequences were performed with a phase array torso/abdomen coil.

**Figure 1:**
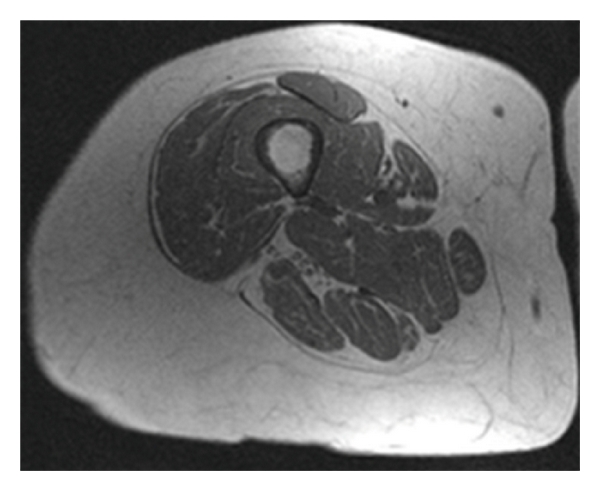
Representative image of mid thigh region showing cross-sectional area of lean and IMAT.

 Four images from the middle 1/3 of each thigh were used to determine average cross-sectional area (cm^2^) of IMAT and lean tissue. Manual tracing eliminated subcutaneous fat and bone and isolated the fascial border of the thigh to create a subfascial region of interest (ROI). Total IMAT and lean tissue were calculated by summing the value of percent fat fraction and percent lean tissue fraction over all pixels within the ROI using custom-written image analysis software (MATLAB; The MathWorks, Natick, Massachusetts). This sum was multiplied by the area of each pixel to give total fat and lean tissue CSAs within the ROI. This method accurately measures fat and lean tissue in pixels that contain both [11] by allowing fractional contributions to the fat and lean tissue CSA calculations. This allows microscopic fat within muscle tissue as well as thin planes of fat adjacent to fascial planes to be accurately taken into account, even when image resolution is inadequate to delineate these visually (Figure 1). 

 The same investigator, blinded to time point of the scan and slice location, performed measurements of individual participants. This technique has demonstrated high levels of intrarater reliability [12], test-retest reliability [13, 14], and concurrent validity when compared to imaging of a cadaveric phantom limb [12].

#### 2.2.3. Mobility Function

Mobility was determined using four tests: (1) a six-minute walk (6MW), (2) stair ascent (Stair A), (3) stair descent (Stair D), and (4) a timed up and go (TUG). These performance tests were chosen to represent mobility function and have been shown to be both valid and reliable in this population [15–17]. The 6MW test, a measure of the distance a subject walks in 6 minutes, was used to assess overall mobility. Participants were asked to cover as much distance as possible in six minutes without running. Distance was recorded in meters. The stair A test required participants to ascend one flight of stairs under close or contact supervision as quickly and safely as possible. Time was recorded to the nearest 0.01 second from a verbal go signal to final foot placement on a standard flight of 10 stairs, and the average of three trials was recorded. The stair D test was performed exactly as the stair A test, except that participants were required to descend one flight of stairs as quickly and safely as possible. The TUG test required participants to rise from a seated position, walk out 3 meters, turn around, and return to sitting as quickly and safely as possible. Time was recorded to the nearest 0.01 second from the time the person's buttocks left the chair until return contact with the chair. Participants were given one practice trial and three test trials. The average of the three test trials was used for statistical analysis.

### 2.3. Statistical Analyses

Data management and statistical analyses were performed with PASW statistics 18.0 (SPSS, Chicago, IL). Descriptive data were calculated for demographic variables and dependent measures and are presented as means ± SD. Pearson correlation coefficients were calculated to determine the bivariate relationship between each muscle and mobility variable. The relative contribution of each muscle variable to explaining the variability in the mobility outcomes were examined using step-wise hierarchical linear regression models. Each mobility test was used as the dependent variable in separate models, with knee extension strength, quadriceps lean tissue, and IMAT considered for entry in a stepwise manner. BMI was entered as a control variable in each model. Criterion for entry to the model was a significance level of *P* < 0.05. For each variable entered in the final model, the part correlation was examined to determine the unique amount of variance in the mobility outcome that was accounted for by the variable. The alpha level was set at <0.05.

## 3. Results

The bivariate correlation of mobility variables with muscle variables revealed moderately strong and significant correlations (*r* range = 0.23–0.55, *P* < 0.05) (Table 2). The direction of the correlations indicates that as strength and average lean tissue increase, mobility function improves. As average IMAT increases, mobility function declines. These results support the use of step-wise regression analyses to examine the unique and shared contributions of the muscle variables towards explaining the variance in mobility measures.

 The multiple regression analysis on the 6MW revealed that the predictors as a group accounted for 34.6% of the variance in walk distance, with strength (*P* = 0.005), IMAT (*P* = 0.002), and lean (*P* = 0.005) each significantly contributing to the final model (*P* = 0.005). The part correlation of strength was 0.23, of IMAT was −0.30, and of lean was 0.23 indicating that strength, IMAT, and lean explained 5.3%, 9.0%, and 5.3% of the variance in the 6MW score, respectively, with all other variables in the model held constant.

 The predictor variables as a group accounted for 45.1% of the variance in stair ascent time, with strength (*P* = 0.001) and IMAT (*P* < 0.001) contributing significantly to the final model (*P* = 0.001). The part correlation of strength was −0.54 and of IMAT was 0.39, indicating that strength and IMAT explained 29.2% and 15.2% of the variance in the stair ascent scores, respectively, with all other variables in the model held constant.

 The predictor variables as a group accounted for 37.4% of the variance in stair descent time, with strength (*P* = 0.001) and IMAT (*P* = 0.001) contributing significantly to the final model (*P* = 0.001). The part correlation of strength was −0.49 and of IMAT was 0.37, indicating that strength explained 24.0% and IMAT explained 13.7% of the variance in stair descent with all other variables in the model held constant.

 The predictor variables as a group accounted for 26.5% of the variance in TUG score, with strength (*P* = 0.001) and IMAT (*P* = 0.001) contributing significantly to the final model (*P* = 0.001). The part correlation of strength was −0.42 and of IMAT was 0.28, indicating that strength and IMAT explained 17.6% and 7.8% of the variance in TUG scores, respectively, with all other variables in the model held constant (Table 3). 

## 4. Discussion

The novel finding in this investigation is that even when accounting for total body mass, thigh muscle adipose tissue surfaces as a potent muscle variable related to the ability of older adults to move about in their community. Specifically, normalized muscle strength, IMAT and lean tissue composition measured via MRI were tested to determine the contribution of these muscle variables to declining mobility levels in older adults. The link between IMAT and mobility deficits has been shown in large epidemiological studies [18]; however, this is the first study to target older individuals needing mobility related rehabilitation interventions.

 Our results are in agreement with previous reports. Skeletal muscle lipid content in older men and women is independently associated with maximal torque production even after adjusting for muscle size, height, weight, age, and race, accounting for 32–36% of the total variance in strength [5]. Greater fat infiltration into muscle is also associated with increased risk of future mobility loss in older men and women [8]. Our results punctuate the potential negative impact of fatty infiltration of muscle on mobility function, an important component of sarcopenia in the elderly and are particularly interesting considering that IMAT explains a surprisingly high amount of the variance in these mobility tasks.

Though by no means causal, the correlations reported here between IMAT and specific performance tests of mobility function in older adults support, in part, the potential inflammatory pathway linking ectopic fat deposition and deteriorating physical ability of older individuals. Evidence of a direct effect of inflammatory cytokines on muscle catabolism [19, 20], combined with the knowledge that inflammation is often elevated with aging [21, 22] suggests inflammation could be a reason for functional decline and frailty. The current study, however, does not provide any data to support this hypothesized role, and the lack of any biochemical data should be considered a limitation. Recent evidence [23, 24] suggests that an elevated inflammatory milieu is associated with loss of muscle, strength, and function in the older individuals, making it tempting to speculate that a connection between IMAT, inflammation, and sarcopenia exists, and future interventions should target not only the impact of lean tissue loss, but also of increased IMAT deposits.

There is wide variation in aging populations with respect to mobility levels, comorbid conditions, and body composition. Because of this, strong correlations between these variables are difficult to identify. A recent review of body composition factors and their relationship to mobility in older individuals [25] highlights this fact, emphasizing the importance of our results and suggesting that future studies should examine IMAT as an important variable with respect to sarcopenia and mobility function in older individuals. Future studies are needed to confirm these findings and to determine whether decreases in IMAT are associated with concurrent improvement in mobility function. Additionally, the participants in this study, while varying in age between 60 and 93 years, were generally high-functioning individuals. Because individuals functioning at lower levels were not included, this should be considered when generalizing these findings.

 This newly identified muscle impairment has not traditionally been targeted by clinicians concerned with sarcopenia, but now in addition to the loss of lean tissue and muscle strength, IMAT should be considered an important factor that may contribute to deficits in mobility in the aging population. Preliminary data show that moderate physical activity in older adults may prevent skeletal muscle fat infiltration [26] and that exercise can decrease IMAT [27, 28]. This suggests that IMAT may be amenable to change with exercise countermeasures, though these findings are limited by small sample sizes and nonrandomized designs. Well controlled, randomized studies are needed to determine the best exercise countermeasure to decrease IMAT and importantly, how this decrease impacts mobility function in mobility limited older adults.

## 5. Conclusion

Thigh IMAT contributes a significant amount of variance to the mobility performance of older adults needing rehabilitation, even when accounting for total body mass. IMAT has a negative impact on mobility function, an important component of sarcopenia in the elderly. Locomotor muscle fatty infiltration, in addition to lean tissue and muscle strength, should be considered an important factor contributing to mobility deficits in this vulnerable population.

## Figures and Tables

**Table 1:** Characteristics of the 109 participants (females *n* = 77, males *n* = 32). body mass index (BMI), cross-sectional area (CSA), six minute walk (6 MW), stair ascent (stair A), stair descent (stair D), timed up and go (TUG).

Variable	Mean (SD)
Age (years)	74.1 (6.8)
BMI (kg∗m^2^)	28.6 (5.6)
Thigh muscle CSA (cm^2^)	97.0 (23.3)
Thigh IMAT CSA (cm^2^)	15.6 (6.7)
Knee extension strength (N)	228.2 (73.2)
6 MW (meters)	409.9 (120.3)
Stair A (seconds)	7.6 (3.7)
Stair D (seconds)	7.2 (4.1)
TUG (seconds)	8.9 (3.4)

**Table 2:** Bivariate correlations between muscle and mobility variables. Intramuscular adipose tissue (IMAT), cross-sectional area (CSA), six minute walk (6 MW), stair ascent (stair A), stair descent (stair D), timed up and go (TUG).

	6 MW (m)	Stair A (s)	Stair D (s)	TUG (s)
Strength (N/BMI)	0.50**	−0.55**	−0.49**	−0.45**
Thigh IMAT CSA (cm^2^)	−0.33**	0.39**	0.36**	0.30**
Thigh muscle CSA (cm^2^)	0.38**	−0.32**	−0.30**	−0.23*

**P < 0.01, *P < 0.05.

**Table 3:** Hierarchical regression results. six minute walk (6 MW), stair ascent (stair A), stair descent (stair D), timed up and go (TUG).

Variable	Regression coefficient (95% CI)	*P*-value	Part correlation	*R* ^2^
6MW				34.6
BMI	−0.15 (−7.5–0.86)	0.12	−0.15	
Strength	0.27 (0.14–0.76)	0.005	0.23	
IMAT	−0.31 (−9.1–−2.20)	0.002	−0.30	
Lean	0.28 (0.44–2.44)	0.005	0.23	
Stair A				45.1
BMI	−0.07 (−0.16–0.07)	0.46	−0.05	
Strength	−0.55 (−0.04–−0.02)	0.001	−0.54	
IMAT	0.47 (0.16–0.36)	0.001	0.39	
Stair D				37.4
BMI	−0.09 (−0.20–0.07)	0.36	−0.07	
Strength	−0.49 (−0.04–−0.02)	0.001	−0.49	
IMAT	0.45 (0.16–0.39)	0.001	0.37	
TUG				
BMI	−0.003 (−0.13–0.12)	0.97	−0.01	26.5
Strength	−0.42 (−0.03–−0.01)	0.001	−0.42	
IMAT	0.34 (0.07–0.28)	0.001	0.28	

## References

[1] Cruz-Jentoft AJ, Baeyens JP, Bauer JM (2010). Sarcopenia: European consensus on definition and diagnosis. *Age and Ageing*.

[2] Goodpaster BH, Park SW, Harris TB (2006). The loss of skeletal muscle strength, mass, and quality in older adults: the Health, Aging and Body Composition Study. *Journals of Gerontology—Series A*.

[3] Marcus RL, Addison O, Kidde JP, Dibble LE, Lastayo PC (2010). Skeletal muscle fat infiltration: impact of age, inactivity, and exercise. *The Journal of Nutrition, Health and Aging*.

[4] Delmonico MJ, Harris TB, Visser M (2009). Longitudinal study of muscle strength, quality, and adipose tissue infiltration. *American Journal of Clinical Nutrition*.

[5] Goodpaster BH, Carlson CL, Visser M (2001). Attenuation of skeletal muscle and strength in the elderly: the health ABC study. *Journal of Applied Physiology*.

[6] Goodpaster BH, Thaete FL, Kelley DE (2000). Thigh adipose tissue distribution is associated with insulin resistance in obesity and in type 2 diabetes mellitus. *American Journal of Clinical Nutrition*.

[7] Hilton TN, Tuttle LJ, Bohnert KL, Mueller MJ, Sinacore DR (2008). Excessive adipose tissue infiltration in skeletal muscle in individuals with obesity, diabetes mellitus, and peripheral neuropathy: association with performance and function. *Physical Therapy*.

[8] Visser M, Goodpaster BH, Kritchevsky SB (2005). Muscle mass, muscle strength, and muscle fat infiltration as predictors of incident mobility limitations in well-functioning older persons. *Journals of Gerontology—Series A*.

[9] Hardin BJ, Campbell KS, Smith JD (2008). TNF-*α* acts via TNFR1 and muscle-derived oxidants to depress myofibrillar force in murine skeletal muscle. *Journal of Applied Physiology*.

[10] Bruunsgaard H, Bjerregaard E, Schroll M, Pedersen BK (2004). Muscle strength after resistance training is inversely correlated with baseline levels of soluble tumor necrosis factor receptors in the oldest old. *Journal of the American Geriatrics Society*.

[11] Kovanlikaya A, Guclu C, Desai C, Becerra R, Gilsanz V (2005). Fat quantification using three-point Dixon technique: in vitro validation. *Academic Radiology*.

[12] Dibble LE, Hale TF, Marcus RL, Droge J, Gerber JP, LaStayo PC (2006). High-intensity resistance training amplifies muscle hypertrophy and functional gains in persons with parkinson’s disease. *Movement Disorders*.

[13] Elder CP, Apple DF, Bickel CS, Meyer RA, Dudley GA (2004). Intramuscular fat and glucose tolerance after spinal cord injury—a cross-sectional study. *Spinal Cord*.

[14] Gorgey AS, Dudley GA (2007). Skeletal muscle atrophy and increased intramuscular fat after incomplete spinal cord injury. *Spinal Cord*.

[15] Enright PL, McBurnie MA, Bittner V (2003). The 6-min walk test: a quick measure of functional status in elderly adults. *Chest*.

[16] Kennedy DM, Stratford PW, Wessel J, Gollish JD, Penney D (2005). Assessing stability and change of four performance measures: a longitudinal study evaluating outcome following total hip and knee arthroplasty. *BMC Musculoskeletal Disorders*.

[17] Shumway-Cook A, Brauer S, Woollacott M (2000). Predicting the probability for falls in community-dwelling older adults using the timed up and go test. *Physical Therapy*.

[18] Visser M, Kritchevsky SB, Goodpaster BH (2002). Leg muscle mass and composition in relation to lower extremity performance in men and women aged 70 to 79: the Health, Aging and Body Composition Study. *Journal of the American Geriatrics Society*.

[19] Garcia-Martinez C, Lopez-Soriano FJ, Argiles JM (1993). Acute treatment with tumour necrosis factor-*α* induces changes in protein metabolism in rat skeletal muscle. *Molecular and Cellular Biochemistry*.

[20] Haddad F, Zaldivar F, Cooper DM, Adams GR (2005). IL-6-induced skeletal muscle atrophy. *Journal of Applied Physiology*.

[21] Bautmans I, Njemini R, Vasseur S (2005). Biochemical changes in response to intensive resistance exercise training in the elderly. *Gerontology*.

[22] Harris TB, Ferrucci L, Tracy RP (1999). Associations of elevated interleukin-6 and C-reactive protein levels with mortality in the elderly. *American Journal of Medicine*.

[23] Nicklas BJ, Hsu FC, Brinkley TJ (2008). Exercise training and plasma C-reactive protein and interleukin-6 in elderly people. *Journal of the American Geriatrics Society*.

[24] Schaap LA, Pluijm SMF, Deeg DJH (2009). Higher inflammatory marker levels in older persons: associations with 5-year change in muscle mass and muscle strength. *Journals of Gerontology—Series A*.

[25] Kidde J, Marcus RL, Dibble L, Smith S, Lastayo P (2009). Regional muscle and whole-body composition factors related to mobility in older individuals: a review. *Physiotherapy Canada*.

[26] Goodpaster BH, Chomentowski P, Ward BK (2008). Effects of physical activity on strength and skeletal muscle fat infiltration in older adults: a randomized controlled trial. *Journal of Applied Physiology*.

[27] Marcus RL, Kidde J, Dibble L, Addison O, LaStayo PC (2008). Intramuscular fat in older adults and the impact of resistance training. *The Journal of Nutrition, Health and Aging*.

[28] Marcus RL, Smith S, Morrell G (2008). Comparison of combined aerobic and high-force eccentric resistance exercise with aerobic exercise only for people with type 2 diabetes mellitus. *Physical Therapy*.

